# Effects of intranasal mometasone furoate on blood pressure in patients with allergic rhinitis 

**DOI:** 10.5414/ALX01764E

**Published:** 2018-09-01

**Authors:** O. Kartal, O. Baysan, M. Gulec, A.Z. Caliskaner, O. Sener, M. Karaayvaz

**Affiliations:** 1 Gulhane Military Medical Academy and Medical School, Division of Immunology and Allergic Diseases, Ankara, Turkey,; 2 Guven Hospital, Clinic of Cardiology, Ankara, Turkey,; 3 Necmettin Erbakan University Meram Medical Faculty, Division of Immunology and Allergy, Konya, Turkey,; 4 Medicana Hospital, Clinic of Pediatric Allergy Ankara, Turkey

**Keywords:** allergic rhinitis, blood pressure, nasal steroid, mometasone furaoate, nasal congestion

## Abstract

Background: Nasal congestion as the main symptom in patients with allergic rhinitis can impair nasal breathing. It causes hypoxia and concomitant sympathetic system activation, which may also lead to increased blood pressure levels in these patients. Objective: We postulated that appropriate therapy, including intranasal steroids, decreases blood pressure levels in patients with allergic rhinitis. Methods: In our study, we investigated the effect of intranasal steroid (4 weeks of mometasone furoate) on blood pressure changes in 45 patients with allergic rhinitis whose main complaint was nasal congestion. We used ambulatory monitoring for determining blood pressure levels before and after intranasal steroid therapy. None of the patients had any other systemic diseases. Results: We found a significant decrease of daytime systolic and diastolic blood pressures and mean blood pressure values (daytime systolic blood pressure: 120 vs. 117 mmHg, p = 0.024; daytime diastolic blood pressure: 73 vs. 71 mmHg, p = 0.027; daytime mean blood pressure: 86 vs. 83 mmHg, p = 0.007). Although insignificant, we also found lower night-time systolic and mean blood pressure values (nighttime systolic blood pressure: 109 vs. 107 mmHg, p = 0.182; nighttime mean blood pressure 77 vs. 73 mmHg, p = 0.116). Conclusions: We found that post-treatment daytime average systolic, diastolic, and mean arterial blood pressure levels were significantly lower compared to values obtained during exacerbation of allergic rhinitis. Decrease in blood pressure with treatment of allergic rhinitis and nasal congestion suggests that nasal congestion and impaired nasal respiration may affect blood pressure and potentially cause serious problems in hypertensive patients with allergic rhinitis.

**German version published in Allergologie, Vol. 38, No. 9/2015, pp. 471-476**

## Introduction 

Allergic rhinitis (AR) is characterized by chronic inflammation of the nasal mucosa and is usually treated with medications targeting symptomatic relief [1]. Unfortunately, nasal congestion and other comorbid diseases, including asthma, sinusitis, or otitis, may lead to chronic impairment and a decreased quality of life in these patients [1, 2]. It is well known that patients with AR cannot sleep “well” and wake up exhausted in the morning [1]. Their cognitive functions are also decreased [3, 4]. It is thought that hypoxemia due to nasal congestion, particularly occurring during night sleep, activates the sympathetic system, causes reflex vasoconstriction, and increases heart rate and blood pressure in patients with AR as in patients with obstructive sleep apnea syndrome (OSAS) [5]. 

The positive effect of AR treatment on blood pressure levels in hypertensive patients was described in a controlled study [6]. In this study, after 8 weeks of AR treatment, systolic blood pressure levels were significantly decreased in hypertensive patients (who were also taking antihypertensive medications). 

We hypothesized that treatment of AR may decrease blood pressure levels in normotensive patients with severe symptoms by improving of nasal congestion and consequent hypoxia. 

## Methods 

**Study participants**

We enrolled 70 consecutive patients aged between 18 and 40 years who were diagnosed with seasonal AR by epidermal allergy tests between March and August, but had not received any medications to treat rhinitis. 

**Skin prick test**

To confirm the allergy, all patients underwent skin prick tests (SPT), as previously reported, with aeroallergens common in Turkey (grass mix, cereals mix, weed mix, tree mix, house dust mite, cat fur, and dog epithelia) (Laboratoires Stallergènes, Antony, France) [1]. 

**Evaluation of patient symptoms**

Daytime sleepiness was evaluated using the Epworth Sleepiness Scale [7]. It consists of 8 self-rated items, each scored from 0–  3, which measure a subject’s habitual “likelihood of falling asleep or dozing” in common situations of daily living. A score of 10 or more is considered as ‘sleepy’. Patients with a score of 10 or more were included in the study. 

The VAS (Visual Analog Scale) was used to assess the subjective feeling of combined nasal symptoms (nasal obstruction, rhinorrhea, sneezing, nasal pruritus, and nasal obstruction); it ranges from 0 (nasal symptoms, not at all bothersome) to 10 (nasal symptoms, extremely bothersome) [8]. 

The patients were re-evaluated after the treatment period, and 24-hour blood pressure monitoring was repeated in patients with VAS score ≤ 3. 

The therapy was accepted as beneficial in patients whose pretreatment VAS score of ≥ 8 decreased to a post-treatment VAS score of ≤ 3. Post-treatment evaluations were performed on patients with VAS scores of 3 or below. 

**Office blood pressure measurement**

Inhospital blood pressure was measured using a mercury sphygmomanometer following a 5-min. rest in a seated position. 

**Ambulatory blood pressure measurement (ABPM)**

Ambulatory blood pressure was measured with Spacelabs 90207 ABPM device (Spacelabs Healthcare Inc. Redmond, Washington, USA), which is an oscillometric device. The device was programmed to measure blood pressure once every 30 minutes beginning at 9:00 a.m. until 9:00 a.m. the day after [9]. 

The patients were informed that they should not change their daily activity and that their arm should be in a comfortable position during the measurement. The procedure was considered successful if 85% of the measurements were read. 

**Patient exclusion**

A detailed patient history, physical examination, and laboratory tests were deemed sufficient to confirm that patients did not have any concomitant disease. 

Twenty-five patients who had mechanical obstruction that affects nasal airflow (i.e., nasal septal deviation or turbinate hypertrophy), patients with perennial symptoms, sensitized to perennial allergens, history of hypertension, any pulmonary or cardiovascular system (CVS) diseases, OSAS, diabetes mellitus or hyper-hypothyroidism, or obesity (BMI > 30 kg/m^2^) were excluded. 

**Figure 1.**
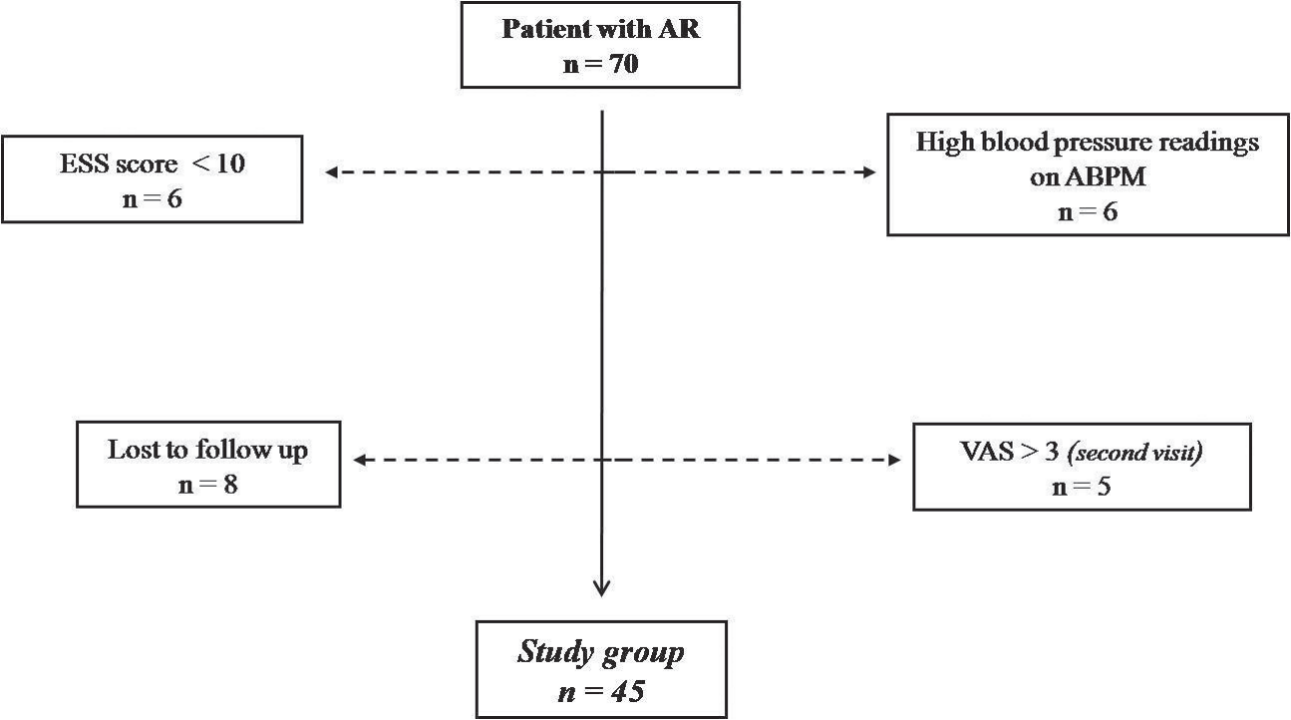
Flow diagram of patient selection. Abbreviations: ESS = Epworth Sleepiness Scale; AR = Allergic rhinitis; ABPM = Ambulatory blood pressure measurement; VAS = Visual analog scale.

High inhospital blood pressure (> 140 – 90 mmHg) or ambulatory blood pressure measurements (> 135 – 85 mmHg) were accepted as valid exclusion criteria. The University Bioethics Committee approved the study protocol, and written informed consent was obtained from each patient. Causes for exclusion are presented in Figure 1. In the end, the study included 45 patients (mean age 29.6 ± 7.0 years, male/female ratio 36/9). 

**Treatment of allergic rhinitis**

In this 4-week study, patients with AR received mometasone furoate nasal spray (Nasonex™), 200 µg each morning. 

**Statistical Evaluation**

The software SPSS 15.0 (Chicago, IL, USA) was used for statistical evaluation. Continuous variables were presented as mean ± standard deviation (SD) according to data distribution. Categorical variables were given as percent value. Data distribution was tested with Shapiro-Wilk test. Paired-samples Student’s t-test was used for continuous variables. c2-test was also used for categorical data. A p value < 0.05 was considered as statistically significant. 

## Results 

We studied 45 subjects (9 females, 36 males; age (mean ± SD) = 29.6 ± 7.0 years) with AR. The mean time since onset of rhinitis was 7.16 years (SD ± 3.8). A history of atopic diseases in the family was reported by 22 patients (48.9%). A history of smoking was observed only in male patients (Table 1). 

The mean values of ESS (Epworth Sleepiness Scale) score and VAS of the patients were decreased after treatment (Table 1). 

After examining the ABPM of the patients before and after intranasal steroid treatment, a significant decrease in systolic and diastolic blood pressure and average blood pressure levels at daytime was detected (daytime systolic blood pressure: 120 vs. 117 mmHg, p = 0.024; daytime diastolic blood pressure: 73 vs. 71 mmHg, p = 0.027; daytime mean blood pressure: 86 vs. 83 mmHg, p = 0.007, respectively) (Table 2). 

On the other hand, no significant difference was detected in night blood pressure values (nighttime systolic blood pressure: 109 vs. 107 mmHg, p = 0.182; nighttime diastolic blood pressure: 65 vs. 65 mmHg, p =0.944; nighttime mean blood pressure 77 vs. 73 mmHg, p = 0.116, respectively) (Table 2). 

We found a gender-specific blood pressure response to intranasal steroid therapy. Daytime blood pressure values were lower after the therapy only in male patients, while the values for female patients did not show any difference (daytime systolic blood pressure in men: 122 vs. 119 mmHg, p = 0.016; daytime diastolic blood pressure in men: 74 vs. 72 mmHg, p = 0.009; daytime mean blood pressure in men: 86 vs. 83 mmHg, p = 0.003 , respectively), (daytime systolic blood pressure in women: 112 vs. 110 mmHg, p = 0.638; daytime diastolic blood pressure in women: 71 vs. 70 mmHg, p = 0.874; daytime mean blood pressure in women: 82 vs. 82 mmHg, p = 0.910 , respectively) (Table 2). 

## Discussion 

We found lower daytime systolic, diastolic, and mean blood pressure levels only in male normotensive patients with AR taking intranasal steroid treatment compared to pretreatment blood pressure measurements taken with ABPM. However, nighttime ambulatory blood pressure levels did not show any statistically significant decrease with intranasal steroid therapy in either male or female patients. 

In a previous study, significant reductions of systolic blood pressure were observed in hypertensive patients treated for AR [6]. Although there are methodological differences between the studies, such as patient characteristics, similar results were obtained in our study. To our knowledge, our study is the first to investigate the potential association between nasal congestion and blood pressure in normotensive patients with AR. 

It is known that nasal respiration is physiological and more effective than mouth respiration for effective alveolar ventilation in healthy people [10]. In contrast, nasal obstruction plays a role in sleep-disordered breathing, and daytime nasal obstruction is an independent risk factor for OSAS [11, 12]. It is generally accepted that there is an increased risk for development of hypertension and adverse cardiovascular events in patients with OSAS [13]. 

We selected patients with an Epworth score in the range of ‘sleepy’ (more than 10), which confirmed the presence of daytime sleepiness in our study. Sleep disturbances are very frequent in AR patients such that only 3.2% of patients have a good quality of sleep [14]. Snoring, obstructive sleep apnea, and/or hypopnea and microarousals have been seen in AR patients [15]. Steavska et al. [16] have suggested that AR is associated with mild forms of OSAS. Although sneezing, rhinorrhea, and nasal pruritus may contribute to sleep problems, nasal congestion seems to be a predominant trigger [5]. Nasal congestion reduces the internal nasal diameter, increases airway resistance to nasal airflow, and results in nasal obstruction. AR patients with nasal congestion have a 1.8 times increased risk for sleep-disordered breathing compared to AR patients without nasal congestion [17]. Ensuing nocturnal awakenings and excessive daytime sleepiness are frequent, especially during AR exacerbations [18]. In addition, inflammatory mediators, such as histamine and cytokines (interleukin-1B, interleukin-4, and interleukin-10), may also affect sleep quality, especially through decreasing the restorative REM sleep period [19]. Allergic rhinitis patients generally experience their symptoms on a 24-hour basis with some variability. Nasal congestion is usually the worst during night- and early-morning hours upon awakening [20]. Likewise, inflammatory mediators show levels peaking in the early morning hours [21]. 

We were not able to detect any statistical difference in nighttime blood pressure levels. Indeed, intermittent hypoxia does not lead to nighttime blood pressure elevation. According to the findings of Tamisier et al. [22], there was no increase in nocturnal catecholamine excretion, and, therefore, no increase in nocturnal blood pressure. Moreover, the same authors claimed that shorter sleep time is directly related to the nondipping pattern of blood pressure. They also suggested that healthy subjects with intermittent nocturnal hypoxia, as in our study participants, continued to exhibit a normal dipping pattern of blood pressure because of normal sleep organization with a significant amount of slow-wave sleep. Interestingly, this was followed by persistent sympathetic hyperactivity during the daytime, which may explain our finding of high blood pressure levels during the daytime [22]. 

We found decreased blood pressure values with the therapy only in male patients (Table 2). It is known that sex hormones cause different, gender-specific cardiovascular system responses to hypoxia at the level of the central nervous system [23]. Previous studies have confirmed that hypoxia-driven cardiovascular system responses in subjects living at highaltitude were attenuated in female subjects compared to males [24, 25]. 

Nasal steroids are considered first-line treatment options when nasal congestion is the predominant symptom. A regular administration of intranasal corticosteroids for 2 – 4 weeks decreases nasal congestion, attenuates sleep disturbance, and thereby improving the quality of life in patients with AR [26, 27]. Therefore, it is reasonable to suggest that nasal steroids may also lessen blood pressure elevations during AR exacerbations. To support this assumption we found lower daytime blood pressure levels after nasal steroid therapy in normotensive AR patients. 

We confined our study population to patients having a relatively high symptom burden (high Epworth Sleepiness Scale and Visual Analog Scale scores), which clearly introduced a selection bias. We thought that patients with more pronounced symptoms should have higher blood pressure values at baseline and also show a noticeable blood pressure response to the therapy. 

The absence of pulse oximetric data confirming presence of hypoxia is another major limitation of our study. However, previous data have already shown that nasal blockage due to allergic inflammation caused hypoxia as measured by pulse oximetry [28]. 

Allergic rhinitis may cause an increase in daytime blood pressure values, especially in male patients. Although this increase may be less in magnitude in healthy normotensive AR patients, it may lead to unexpected clinical events, such as hypertensive crisis, in AR patients who already have hypertension. 

## Conflict of interest 

There was no financial support for the study. The authors have no conflict of interest to declare. 

**Table 1.** Patient demographics and characteristics^1^.

Variable	Finding (n = 45)
Age, mean ± SD, years	29.6 ± 7.0
Sex, No. (%) Male Female	36 (80) 9 (20)
Smoker, n (%)	9 (20)
Family history of atopy, n (%)	22 (48.9)
Family history of hypertension, n (%)	20 (44.4)
BMI, mean ± SD, (kg/m2)	24.6 ± 2.0
Duration of allergic rhinitis, mean ± SD, y	7.16 ± 3.8
VAS (pretreatment), mean ± SD, (cm)	8.53 ± 0.6
VAS (post-treatment), mean ± SD, (cm)	1.57 ± 0.6
Epworth sleepiness scale score, mean ± SD, (pretreatment)	14.27 ± 3.3
Epworth sleepiness scale score, mean ± SD, (post-treatment)	1.44 ± 1.3

BMI: Body Mass Index, VAS: Visual Analog Scale. ^1^Data are presented as number (percentage) or mean ± SD unless otherwise stated.

**Table 2.** Blood pressure levels in patients with AR: comparison between MFNS^1^ a before and after treatment.

**Variable**	**Mean value ** **before treatment (mmHg)**	**Mean value ** **after treatment (mmHg)**	**p value**
Average systolic BP at daytime All patients Male Female	120 ± 9.1 122 ± 8.6 112 ± 6.2	117 ± 9.4 119 ± 7.7 110 ± 12.2	0.024 0.016 0.638
Average diastolic BP at daytime All patients Male Female	73 ± 5.5 74 ± 5.7 71 ± 3.7	71 ± 7.4 72 ± 7.2 70 ± 8.7	0.027 0.009 0.874
Average systolic BP at nighttime	109 ± 8.4	107 ± 10.3	0.182
Average diastolic BP at nighttime	65 ± 6.4	65 ± 9.3	0.944
Average BP at daytime All patients Male Female	86 ± 5.7 86 ± 5.7 82 ± 4.7	83 ± 7.6 83 ± 7.0 82 ± 9.9	0.007 0.003 0.910
Average BP at nighttime	77 ± 8.0	73 ± 16.2	0.116

BP: blood pressure; MFNS: mometasone furoate nasal spray. ^1^Data are presented as mean ± SD
